# Anxiety and depression in geriatric hemodialysis patients: factors that influence the border of diseases

**DOI:** 10.3389/fpsyg.2023.1281878

**Published:** 2023-11-24

**Authors:** Brunilda Elezi, Erjona Abazaj, Bruno Zappacosta, Malvina Hoxha

**Affiliations:** ^1^Department of Clinic, Faculty of Medical Technical Science, University of Elbasan Aleksander-Xhuvani, Elbasan, Albania; ^2^Department of Epidemiology and Infectious Diseases Control, Institute of Public Health, Tirana, Albania; ^3^Department for Chemical-Toxicological and Pharmacological Evaluation of Drugs, Catholic University Our Lady of Good Counsel, Tirana, Albania

**Keywords:** hemodialysis patients, prevalence, anxiety, depression, risk factors

## Abstract

**Introduction:**

The two main psychological issues that people with end-stage renal disease (ESRD) experience are depression and anxiety. We conducted this study to determine both the prevalence of depression and anxiety, and the factors associated with them, among hemodialysis patients.

**Methods:**

Patients aged 18 years or older, who had received hemodialysis in a hemodialysis center in Elbasan, for at least 3 months were included in this study. Beck Anxiety Inventory and Beck Depression Inventory Instruments were used to assess hemodialysis patients levels of depression and anxiety.

**Results:**

Overall, 107 hemodialysis patients (men 65.4%) with a mean age of 57 ± 8.9 years were enrolled in the study. The prevalence of anxiety and depression resulted to be 85.98, and 84.11%, respectively. We found a significant difference in depression and anxiety scores in patients age groups of 61–70 years old (OR = 1.8; 95% CI [0.7–3.7]; *p* = 0.041), in non-smoking patients (OR 3.4; 95% CI [1.09–8.2]; *p* = 0.04), in diabetic patients (OR 3.4; 95% CI [1.09–8.2]; *p* = 0.04), and in patients with a time in dialysis of 6–10 years and >11 years, respectively, (OR 3.4; 95% [1.5–9.0]; *p* = 0.01), (OR1.3; 95% CI [0.4–3.6]; *p* = 0.04).

**Conclusion:**

Our study shows that the prevalence of mental disorders (depression and anxiety) is high among patients with ESRD on maintenance hemodialysis. We recommend a routine screening and referral to psychological health specialists to evaluate the mental health disorders among hemodialysis patients with the primary aim of improving their quality of life.

## 1 Introduction

Chronic kidney disease (CKD) is a major non-communicable disease with gradual deterioration that affects over 10 % of the world’s population, or over eight hundred million patients (Kovesdy, 2011). Furthermore this silent killer is more common in geriatric ages, females, and those suffering from metabolic syndrome (Clemens et al., 2019). Over the last decades, the CKD global burden is increasing and is assumed to become the fifth foremost cause of years of life lost by 2040 (Foreman et al., 2018). Aside from the disease burden, CKD has a significant impact on individuals, as well as health care systems (Kampmann et al., 2023), since it is associated with negative clinical and economic outcomes (Bello et al., 2017).

Due to the lack of treatment and the duration of the disease, many of these patients suffer gradual deterioration and end up with end-stage renal disease (ESRD), as consequence, patients undergo the hemodialysis (HD) process. On the other hand, going to the hospital to undergo HD process many times per week, considering the long duration of the process, the weariness both for the patients, and for the families who accompany the patient, and, most importantly, the expensive cost, contribute to the loss of hope for better health. All these factors can bring to mental health problems, such as anxiety and depression. Fernandez et al., reported that if the prevalence of depression and anxiety is compared between the general population and HD patients, the latter will definitely have a greater prevalence of these two mental diseases (Fernandez et al., 2022). Various studies found a different incidence of depression among dialysis patients (Dong et al., 2016; Bazazzadeh et al., 2023). Based on data from the World Health Organization (WHO), worldwide prevalence rates of depression and anxiety in 2015 have been determined to be 4.4 and 3.6%, respectively (World Health Organization, 2017). A Lancet study estimated that after adjustment for COVID-19 pandemic the worldwide prevalence of anxiety disorders was 374 million people, versus 298 million people before adjustment for COVID-19 (COVID-19 Mental Disorders Collaborators, 2021). In addition, the worldwide prevalence of anxiety and depression has increased by 25% in the first year of COVID-19 pandemic (World Health Organization, 2022), in confront to the global incidence of depression that was estimated to be over 18% between 2005 and 2015 (World Health Organization, 2017). The projected global incidences of depressive disorders and anxiety among patients with kidney disease, and end-stage renal disease are not precise, and range from 0 to 100%, based on the criteria used for diagnosis, on the measurement technique, and characteristics of the population (Kimmel and Peterson, 2005; de Brito et al., 2019). In addition, in Albania 3.8, and 4.8% of the population is reported to suffer from anxiety disorders and depression, respectively (World Health Organization, 2017), however no data are reported on anxiety and depression among patients with kidney disease, and end-stage renal disease in Albania.

People on dialysis, particularly those in the final stages (ESRD), are constantly battling this disease, as well as other comorbidities. On the other hand, they also have particular psychological conditions due to various socio demographic factors (Turkistani et al., 2014; Ye et al., 2022), which may contribute to the occurrence, or severity of mental health diseases such as depression and anxiety (Loosman et al., 2015; Gerogianni et al., 2018), having a significant impact on their expected health outcomes, or change in quality of life (Huang et al., 2021; Zhang et al., 2023). Shirazian et al. (2016) reported that depression is connected with a higher risk of hospitalization, an increased number of fatalities from any factor in these patients (Kimmel et al., 2019), a lack of appetite, and poor compliance with the dialysis process (Skoumalova et al., 2020; Cheng et al., 2022). Anxiety, on the other hand, has been linked to an increase in co-morbidities, a longer time of hospitalization, and a decrease in vitality (Nagy et al., 2023). In addition, different studies have assessed the role of socio-demographic variables in hemodialysis patients, reporting that age, gender, residence, economic, job and marital status, influence the quality of life of hemodialysis patients (Anees et al., 2014; Togay and Akyüz, 2023). Tsay and Healstead (2002) depression and self-care efficacy are significant predictors of quality of life of HD patients after controlling for the age effect. In line with these findings, Bai et al. (2015) reported that increased age, lower physical activity status, unemployment increased the depression in HD patients. Most clinicians neglect the psychological problems of depression and anxiety among dialysis patients, particularly those in the final stages of their disease (Qawaqzeh et al., 2023). A deeper understanding of the prevalence of these disorders, as well as the factors that contribute to the severity of these diseases in hemodialysis patients, is required.

We conducted this study to determine the prevalence of depression and anxiety, as well as the factors associated with them, among hemodialysis patients, as well as the way depression and anxiety have affected their quality of life. The aim of this study is to assess the socio-demographic features and comorbidities of patients having hemodialysis in an Elbasan dialysis center, the prevalence of depression and anxiety disorders among these patients, the factors that are associated to depression and anxiety in these patients.

## 2 Materials and methods

### 2.1 Design of the study

This study took place in the hemodialysis center in Elbasan City. In this center, 140 patients were treated and underwent hemodialysis. We conducted a cross-sectional study over 3 months (February to April 2023). The questionnaires were filled for each patient after gaining permission and approval from the management staff of this center, with the help and assistance of the nursing staff who had direct contact with these patients. Patients agreement was required to participate in this investigation. Furthermore, the patients were told that their data would be kept confidential and utilized just for the purposes of this study, and that they might withdraw from the study at any time. Based on the Helsinki Declaration, we guaranteed each participant to protect the life, health, dignity, integrity, right to self-determination, privacy, and confidentiality (World Medical Association, 2013). We decided to have the nursing staff conduct this questionnaire because dependability is stronger between nursing personnel and HD patients, in confront to an outsider interviewer, as well as to guarantee the correct gathering of the data and to avoid the incompleteness of each query as much as possible. The selection criteria for this study were all patients over 18 years old, regardless of gender, who agreed to participate in this study. The exclusion criteria were patients under 18 years old, those who did not agree to be part of the study, as well as patients who had undergone hemodialysis for less than 3 months.

### 2.2 Questionnaires and instruments used

This study was designed in two distinct questionnaires by the researchers. Baseline data on the demographic and health-related variables were used in all analyses. Primary socio-demographic explanatory factors in our research were the family’s monthly income, age, gender, dwelling area, education level, living status, and lifestyle (smoking and BMI). Age as a continuous variable was reported in the seven-level ratio scale from18–30, 31–40, 41–50, 51–60, 61–70, 71–80 years, until over 80 years old. Gender, living area, and living status were classified as dichotomous, nominal variables and were presented as man/woman, rural/urban area, and living alone/living with family, respectively. The educational level, taking into account both the number of educational years and the type of education/degree was classified as a categorical ordinal variable. Education was classified into four classes based on the no formal education, elementary/middle school, and high school variable containing information on both the basic education (from elementary school’ to ‘matriculation examination’) and the highest level of education or degree. Monthly income as a continuous variable within a specific range from very low (less than 20.000 lekë/monthly) to very high amounts (more than 40.000 lekë/monthly). In living habits, we included body mass index and smoking habits. BMI as an independent categorical ordinal variable was ordered into four categories: underweight less than18.5 kg/m^3^, normal weight > 18.5- < 25 kg/m^3^, overweight: ≥25 kg/m^3^- < 30 kg/m^3^, obese: ⩾30 kg/m^3^. Smoking status was assessed using only one question “Do you smoke?” The outcome variable of the responders was classified as non-smokers if they had not smoked during their lifetime, and/or if they had smoked during their lifetime or if they are still smoking currently.

Participants hemodialysis features and comorbidities were also investigated, including dialysis time, frequency, and the existence of comorbid disorders such as diabetes mellitus (Tips I and II), cardiovascular disease, hepatitis, or other diseases. We additionally evaluated the patients’ diabetes history and the possibility that any patient has undergone a kidney transplant in the past. The Beck Anxiety Inventory (BAI) and Beck Depression Inventory (BDI) are two regularly used instruments in hemodialysis patients that assess participants’ levels of depression and anxiety (Beck and Steer, 1961; Beck et al., 1988a,b).

BAI and BDI were found to be reliable and valid in Albania with a Cronbach alpha of 0.81 for BAI, and 0.80 for BDI, respectively. In hemodialysis patients, cut-off scores of ≥8 for BAI (Najafi et al., 2016), and ≥75 for BDI (King-Wing Ma and Li, 2016; Saglimbene et al., 2017; El-Magd et al., 2023) were associated with the preferable balance of sensitivity and specificity in end-stage kidney disease patients. BAI scale with 21 items and the scoring is presented as: Not at all; Mildly, but it did not bother me much; Moderately–it wasn’t pleasant at times; Severely–it bothered me a lot. The questions were scored 0123, and the total score is calculated by finding the sum of the 21 items. A score of 0–21 indicates low anxiety; a score of 22–35 indicates moderate anxiety; a score of 36 and above indicates potentially concerning levels of anxiety (Beck and Steer, 1993; Loosman et al., 2010).

The BDI is a 10-item scale that assesses depression-related attitudes and symptoms. The BDI’s internal consistency ranges from 0.73 to 0.92, with a mean of 0.86 (Beck et al., 1988a,b). When the test is scored, each response is assigned a number ranging from 0 to 3, and the entire score is compared to a key to indicate the severity of the depression. The following are the standard cut-offs: A score of 0–4 shows no depression; a score of 5–9 suggests mild depression; a score of 10–14 indicates moderate depression; a score of 15–19 indicates fairly severe depression; and a score of 20–27 indicates severe depression (Beck et al., 1988a,b; Mosleh et al., 2020). Every month, 140 patients are treated for hemodialysis at the institution. Only 107 patients participated in this study and completed all the questionnaire. Thirty-three patients either declined to participate in the study or filled out the questionnaire incorrectly.

### 2.3 Statistical analysis

The data of 107 participants were analyzed using Statistical Package for Social Sciences (SPSS version 26). Standard deviations and mean values are used for displaying continuous variables, whereas frequencies and percentages are used to present categorical variables. Prevalence rates of depression and anxiety were measured (Manea et al., 2012; Najafi et al., 2016). To determine the significance of categorical variables, the Chi-squared test was performed (Nagy et al., 2023). Univariate and multivariate linear regression analyses were used to discover the most significant associated factors of anxiety, and depression. Univariate binary logistic regression analysis was first performed within dependent variables (continuous, dichotomous and nominal scale), with variables found to be significant (*p* < 0.05) included in the subsequent binary multiple logistic regression analyses, to explore independent predictors of depression or anxiety (Maldonado and Greenland, 1993). We confirmed the associations between variables via multivariate analysis. The multivariate analysis findings were displayed as beta, standard error, *p*-value, adjusted odds ratio (AOR), and 95% confidence interval (95% CI) were computed and reported where appropriate (Khan et al., 2019). Statistical significance was defined as a value of *p* of 0.05.

## 3 Results

### 3.1 Socio-demographic variables and comorbidities

The socio-demographic information of participants are shown in Table 1. Patients aged 51–60 and 61–70 years old were majorly present in the hemodialysis center, resulting in 30.8% (33/107), and 27.1% (29/107) of the total, respectively. Additionally, men likewise had the highest number of instances, with 65.4% (70/107) compared to 34.6% (37/107) for women. Approximately 52.3% (56/107) of the patients reported living in rural areas, compared to 47.7% (51) of the remaining patients who lived in urban areas. In contrast to patients with elementary and middle school [32.7% (35/107)], or high school education level [51.4% (55/107)], patients with the highest level of education (university level) 9.3% (10/107), and those with no formal education 6.5% (7/107), were enrolled less in the hemodialysis center.

**Table 1:** Patients’ socio-demographic characteristics.

Variables		Frequency	Percentage
Age groups (years)	18–30 years	4	3.7
	31–40 years	7	6.5
	41–50 years	14	13.1
	51–60 years	33	30.8
	61–70 years	29	27.1
	65–74 years	8	7.5
	71–80 years	9	8.4
	Over 80 years	3	3.0
Gender	Women	37	34.6
	Men	70	65.4
Living area	Rural	56	52.3
	Urban	51	47.7
Education level	No formal education	7	6.5
	Elementary and Middle School	35	32.7
	High school	55	51.4
	University Studies	10	9.3
Living status	Living alone	13	12.1
	Living with family	93	87.9
Monthly income for the family	Low (less than 20.000 lekë/monthly)	32	29.9
	Moderate (between 20.000–40.000 lekë/monthly)	58	54.2
	High (more than 40.000 lekë/monthly)	17	15.9
Lifestyle	Body mass index		
BMI underweight	Less than18.5 kg/m^3^	8	7.5
BMI normal weight	>18.5- < 25 kg/m^3^	53	49.5
BMI overweight	≥25 kg/m^3^- < 30 kg/m^3^	31	29
BMI obesity	≥30 kg/m^3^	15	14
Smoking habits	No	76	71
	Yes	31	29

Only 12.1% of participants (13/107) lived alone, compared to 87.9% (93/107) who lived with their families. The expenditures associated with this illness are rather considerable, and patients were questioned about family incomes. We have divided the monthly income in lekë (lek is the unit of currency in Albania) into three groups based on various economic levels of the nation. Patients with very low incomes (less than 20,000 lekë per month) are included in the first category. Patients with families earning between 20,000 and 40,000 lekë per month are included in the second category. Patients with high incomes (more than 40,000 lekë per month) are included in the third category. The majority of patients, 54.2% (58/107), indicated that their income was moderate based on this split of income level. 29.9% (32/107) of patients referred low-income, while 15.9% (17/107) of patients referred high-income (Table 1).

In terms of lifestyle, we considered only smoking habits and body mass index of patients classified in 4 categories, the first category included all patients with BMI less than 18.5 kg/m3 (underweight), in which 7.5% (8/107) of patients were found; the second category included patients with BMI >18.5–25 kg/m^3^ (normal weight), in which approximately half of the patients were present [49.5% (53/107)]; and the third category included patients with BMI 25 kg/m^3^-30.

Table 2 shows the comorbidities that people on dialysis have had throughout their lives. We assessed the duration of time that these individuals received hemodialysis. Patients enrolled in this study ranged in age from a few weeks old to more than 20 years old. Nearly half of the patients, or 46.7% (50/107), received dialysis for a brief period of time, ranging from 3 months to 5 years. Patients aged 6 to 10 years old represented 29% of the patients (31/107), in respect to patients aged 11 years that represented 24.3% of the total (26/107). Regarding the frequency of the dialysis procedure, 12.0% (12/107) of the patients underwent it once, or twice a week.

**Table 2:** Characteristics of dialysis in participants.

Dialysis and comorbidities		Frequency	Percentage
Time of dialysis (3 months to more than 11 years)	0-month to 5 years	50	46.7
	6–10 years	31	29
	≥11 years	26	24.3
Dialysis frequency	1 or 2 times	12	11.2
	3 times	59	55.1
	4 times	28	26.2
	More than 4 times	8	7.5
Comorbidities (*N* = 76)	Diabetes mellitus	24	31.6
	Arrhythmias	22	30
	Arterial hypertension	21	27.6
	Heart failure	5	6.6
	Hepatitis	2	2.6
	Lupus erythematosus	2	2.6
Diabetes duration (*N* = 24)	1–3 years	3	12.5
	4–5 years	10	41.7
	More than 5 years	11	45.8
Kidney transplant	No	100	93.5
	Yes	7	6.5

More than half of patients, 55.1% (59/107), received hemodialysis three times weekly. Patients who underwent hemodialysis four times or more per week represented 26.2% (28/107), and 7.5% (8/107) of the total population, respectively. Only 71% (76/107) of patients reported having co-morbidities, meaning they had at least one illness besides the kidney issue. Forty-eight patients had cardiovascular disorders, of whom 30% (24/76) had arrhythmias, 27.6% (21/76) had arterial hypertension, and 6.6% (5/107) had heart failure. Of these, 31.6% (24/76) were diabetics with Type II and Type I diabetes. In addition, 2.6% (2/76) of the population experienced either hepatitis, or lupus erythematosus. A total of 24 patients with diabetes mellitus were asked to indicate the year of diagnosis of diabetes. Furthermore, 12.5% (3/24) of patients with this condition have had it for less than 3 years, 41.7% (10/24) have had it for 4–5 years, and 45.8% (11/24) suffered diabetes for more than 5 years. It is important to note that only 6.5% (7/107) of the patients underwent a transplant of one of their kidneys.

### 3.2 Prevalence of depression and anxiety

**Figure 1:**
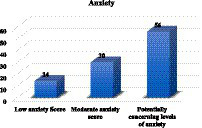
Level of anxiety among hemodialysis patients.

We assessed the participants’ current circumstances with reference to their levels of anxiety and depression in the second part of the questionnaire. Among the 107 patients who agreed to complete the questionnaire, the prevalence of anxiety resulted to be 85.98% (92/107), while the prevalence of depression was 84.11% (90/107). Based on the score calculation, 14% (15/107) scored between 0 and 21 and were classified as having low anxiety, 30% (32/107) scored between 22 and 35 and were classified as having moderate anxiety, and 56% (60/107) scored 36 or higher and were classified as having potentially concerning levels of anxiety (Figure 1).

**Figure 2:**
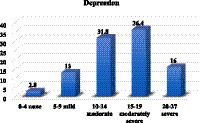
Level of anxiety among hemodialysis patients.

Regarding the severity of depression among hemodialysis patients, 2.8% (3/107) were classified as having none, 13% (14/107) as having mild depression, 31.8% (34/107) as having moderate depression, 36.4% (39/107) as having moderately severed expression, and 16% (17/107) as having severe depression (Figure 2).

### 3.3 Relations between socio-demographic variables and depression/anxiety

In the univariate analysis, we looked at all of the patients’ sociodemographic data, as well as to information about their dialysis or co-morbidities, including anxiety and depression. Tables 3, 4 report the results of the univariate analysis.

**Table 3:** Univariate analysis between sociodemographic data and mental health disorders (anxiety and depression).

Variables		Total number	Frequency of anxiety (92)	Univariate analysis (95%CI)	*P-*value	Frequency of depression (90)	Univariate analysis (95%CI)	*P-*value
Age groups (years)	18–30 years	4	2	Reference		3	Reference	
	31–40 years	7	5	2.1[0.17–3.2]	0.41	5	0.8[0.1–3.6]	0.7
	41–50 years	14	11	3.2[0.25–5.8]	0.22	9	0.6[0.04–4.3]	0.6
	51–60 years	33	30	3.7[0.21–6.4]	0.04	28	1.8[0.2–4.3]	0.5
	61–70 years	29	27	13.5[5.6–19.7]	0.03	26	2.5[1.3–5.0]	0.4
	65-74 years	8	6	1.1[0.03–2.5]	0.4	8	0	
	71–80 years	9	8	0.9[0.01–2.2]	0.2	9	0	
	Over 80 years	3	3	0.0	0	2	0.11[0.02–1.8]	0.8
Gender	Women	37	30	Reference		31		
	Men	70	62	1.8[0.5–4.6]	0.3	59	1.25[04–3.7]	0.7
Living area	Rural	56	49	1.3[0.4–3.9]	0.6	47	1.0[0.4–2.9]	0.7
	Urban	51	43	Reference		43		
Education level	No formal education	7	6	2.5[0.2–3.7]	0.4	5	0.4[0.07–2.6]	0.5
	Elementary and Middle School	35	31	3.3[0.6–7.3]	0.1	30	1.5[0.3–3.8]	0.6
	High school	55	48	2.9[0.4–5.8]	0.17	47	2.1[0.17–3.2]	0.7
	University	10	7	Reference		8	Reference	
Living status	Living alone	13	11	Reference		9	Reference	
	Living with family	93	81	1.2[0.4–6.3]	0.8	81	3.1[0.8–7.2]	0.01
Lekë/Monthly income for the family	Low (less than20.000)	32	29	Reference		30	Reference	
	Moderate (between 20.000–40.000)	58	49	0.5[0.14–2.5]	0.4	45	4.3[0.9–10.7]	0.04
	High (more than 40.000)	17	14	0.4[0.08–2.8]	0.4	15	2.0[0.5–6.4]	0.5
Lifestyle	Body mass index							
BMI underweight	Less than 18.5 kg/m^3^	8	7	Reference		6	Reference	
BMI normal weight	>18.5- < 25 kg/m^3^	53	46	0.9[0.09–8.8]	0.9	44	1.6[0.3–4.9]	0.4
BMI overweight	≥25 kg/m^3^- < 30 kg/m^3^	31	26	0.74[0.08–4.5]	0.7	29	3.2[0.5–8.9]	0.02
BMI obesity	≥30 kg/m^3^	15	13	0.9[0.07–3.9]	0.8	11	1.0[0.1–5.6]	0.9
Smoking habits	No	76	63	2.9[0.63–8.7]	0.01	63	2.9[0.53–8.7]	0.01
	Yes	31	29	Reference		27	Reference	

**Table 4:** Univariate analysis between dialysis and comorbidities data and mental health disorders (anxiety and depression).

Variables		Total number	Frequency of anxiety (92)	Univariate analysis (95%CI)	*P-*value	Frequency of depression (90)	Univariate analysis (95%CI)	*P-*value
Time in dialysis	0-month to 5 years	50	45	Reference		38	Reference	
	6–10 years	31	26	1.5[0.1–2.9]	0.04	29	4.5[0.9–12.5]	0.005
	≥11 years	26	21	0.5[0.3–2.2]	0.3	23	2.4[0.8–7.5]	0.02
Dialysis frequency	1 or 2 times	12	9	Reference		10	Reference	
	3 times	59	49	1.6[0.4–5.7]	0.5	47	1.7[0.9–3.2]	0.5
	4 times	28	27	4.1[0.7–9.3]	0.07	26	2.1[0.3–3.7]	0.3
	More than 4 times	8	7	2.3[1.4–8.7]	0.5	7	1.4[0.1–6.4]	0.7
Comorbidities (*N* = 76)	Diabetes mellitus	24	22	9.1[3.7–16.4]	0.01	20	5.1[2.7–11.6]	0.03
	Arrhythmias	22	19	6.3[2.1–11.4]	0.02	21	1.2[0.6–3.0]	0.08
	Arterial hypertension/cardiovascular diseases	21	18	4.5[1.9–10.5]	0.03	19	2.5[1.08–3.8]	0.1
	Heart failure	5	5	0	0	4	0.4[0.01–1.9]	0.4
	Hepatitis	2	2	0	0	1	1	
	Lupus erythematosus	2	1	Reference		1	Reference	
Diabetes duration (*N* = 24)	1–3 years	3	1	Reference		1	Reference	
	4–5 years	10	7	2.6[0.3–3.7]	0.3	6	1.6[0.4–2.9]	0.4
	More than 5 years	11	8	3.0[1.5–6.2]	0.2	9	4.7[1.8–7.2]	0.01
Kidney transplant	No	100	85	2.2[0.4–7.2]	0.4	84	0.9[0.1–3.4]	0.08
	Yes	7	5	Reference		6	Reference	

Patients between 51 and 60 years old, and those between 61 and 70 years old had a higher chance of having anxiety disorders than people in other age groups; in both cases, the value of *p* was less than 0.05. When the age group is examined in relation to depression, the opposite occurs. We did not observed a significant association between these two age groups; the value of *p* was >0.05 despite the fact that these two age groups had a higher risk than other age groups. Men are more likely than women to experience anxiety and depression, but there are no appreciable disparities between the genders. Univariate analysis revealed significant differences between depression and patients who live with family members (*p* = 0.01), moderate monthly income and depression (*p* = 0.04), and patients with an overweight BMI (*p* = 0.02). An important association between smoking, and both anxiety and depression was found, with value of *p* of 0.01 and 0.02, respectively (Table 3).

Furthermore, in the univariate analysis of the data on the dialysis process and the comorbid diseases that accompany it, a significant relationship is observed between anxiety and depression in patients undergoing hemodialysis for 6–10 years; moreover, depression was significant in patients undergoing hemodialysis for over 11 years. A significant relationship with anxiety is also observed for patients with diabetes, or those having arrhythmia and arterial hypertension, while depression is observed in patients with diabetes, and those with more than 5 years of diabetes. In all the above-mentioned cases, the value of *p* was <0.05 (Table 4).

A more thorough statistical analysis using logistic regression was performed on the variables that showed a greater potential to be significant risk factors for anxiety and depression. A relationship between anxiety disorders and patients’ age groups of 61–70 years was shown using logistic regression analysis (OR = 1.8; 95% CI [0.7–3.7]; *p* = 0.041), no smoking patients (OR 3.4; 95% CI [1.09–8.2]; *p* = 0.04), diabetes (OR 3.4; 95%CI [1.09–8.2]; *p* = 0.04), arrhythmia (OR1.3; 95%CI [0.1–3.5]; *p* = 0.045), and arterial hypertension (OR1.8;95% [0.9–2.7]; *p* = 0.045). On the other hand, the correlations were observed between depression and participants living with their families (OR 3.1; 95% CI[0.8–7.2]; *p* = 0.01), in overweight BMI patients (OR 2.07; 95% CI [0.9–3.5]; *p* = 0.04), in no smoking patients (OR 2.2; 95% CI [0.1–6.9]; *p* = 0.035), in patients in dialysis from 6–10 years and >11 years (OR 3.4;95% [1.5–9.0]; *p* = 0.01), (OR1.3; 95% CI [0.4–3.6]; *p* = 0.04) respectively, and in patients with diabetes duration more than 5 years (OR 1.6; 95%CI [0.9–2.4]; *p* = 0.03) (Table 5).

**Table 5:** Logistic regression for some of the most significant risk factors.

Variables		Anxiety			Depression		
		*β*	Adjust OR (95%CI)	*P-*value	β	Adjust OR (95%CI)	*P-*value
Age	51–60 years	0.02	1.1[0.3–2.8]	0.49	0.501	0.58[0.01–1.4]	0.8
	61–70 years	0.101	1.8[0.7–3.7]	0.041	0.101	0.794[0.2–2.05]	0.5
Living status	Living with family	0.301	0.39[0.01–1.03]	0.9	0.186	3.1[0.8–7.2]	0.01
Monthly income for the family	Moderate (between 20.000–40.000)	0.912	0.75[0.32–1.94]	0.6	−0.004	1.09[0.3–4.5]	0.051
Lifestyle	BMI overweight	0.003	0.23[0.02–1.4]	0.9	0.160	2.07[0.9–3.5]	0.04
	No smoking habits	0.057	1.3[0.5–2.9]	0.03	0.08	2.2[0.1–6.9]	0.035
Time in dialysis	6–10 years	0.07	0.9[0.5–3.2]	0.06	0.0024	3.4[1.5–9.0]	0.01
	≥11 years	0.274	1.1[0.6–3.0]	0.7	0.513	1.3[0.4–3.6]	0.04
Comorbidities	Diabetes mellitus	0.002	3.4[1.09–8.2]	0.04	0.346	0.9[0.05–2.16]	0.048
	Arrhythmias	0.468	1.3[0.1–3.5]	0.04	0.843	0.5[0.03–2.3]	0.2
	Arterial hypertension/cardiovascular diseases	0.812	1.8[0.9–2.7]	0.045	0.034	0.9[0.08–2.4]	0.4
Diabetes duration	More than 5 years	0.138	1.0[0.02–1.9]	0.5	0.019	1.6[0.9–2.4]	0.03

OD, odds ratio; *p-*value < 0.05 = statically significance.

## 4 Discussion

The two main psychological issues that people with ESRD encounter are depression and anxiety. Eventhough the psychiatric diagnostic has drawn great interest as a reliable indicator of psychopathology in ESRD populations, there is still much work to be done, particularly for developing and undeveloped nations (Cukor et al., 2008). The hemodialysis process negatively affects the social, financial, and psychological well-being of all patients (Ginieri-Coccossis et al., 2008). Furthermore, patients with end-stage renal failure go through major changes and adjustments in terms of their personal, professional, and social lives (Kimmel, 2001; Sharma et al., 2022). According to Kimmel (2001) and Goyal et al. (2018), hemodialysis patients must adjust their lifestyle and personal changes in sexual interest and behavior. All of these changes contribute to stress, impacting the patient’s mental health and quality of life (Elezi et al., 2023).

Sociodemographic characteristics, comorbidities, frequency and time of dialysis are widely documented and have an essential influence on hemodialysis patients’ quality of life. All of this result in apparent behavioral changes, as well as in an increase in the burden of mental health. A previous study on hemodialysis patients in Albania found that gender, age, residence area, education level, BMI, monthly income, alcohol use, the origin of renal disease, time of dialysis, frequency of dialysis, and the presence of comorbidities all have an impact on HD patients’ quality of life (Elezi et al., 2023). Most studies found that the methods used for diagnosis, as well as age, residence, education, marital status, insufficient finances, employment, malnutrition, sexual activity, comorbidity, were associated with mental health issues in end-stage kidney disease patients (Picariello et al., 2017; Traisathit et al., 2019). In addition, depression and anxiety were found to be substantially linked with gender, low level of education, higher patient age, retirement, bad financial condition, marital status, and co-morbidities in Greek hemodialysis patients (Gerogianni et al., 2018). On the other hand, Kao et al. (2020) did not fond a significant relationship between anxiety, depression, and socio demographic characteristics such as marital status, gender, age, academic achievement, work status, and the number of comorbidities.

In Albania, depression and anxiety are underdiagnosed in patients with ESRD. To our knowledge, this is the first study to investigate mental health disorders, such as depression and anxiety among hemodialysis patients in Albania. We have conducted this study to determine the prevalence and the factors associated with depression and anxiety among hemodialysis patients, as well as the association between socio-demographic variables and depression and anxiety among these patients.

The minimum age in this study was 18 years old, and the greatest age was 86 years old, with a mean age of 57 ± 8.9. The majority of the hemodialysis participants in our study were elder patients. Consequently, up to 71.02% of patients had concurrent chronic illnesses, such as diabetes, cardiovascular disease, arterial hypertension, hepatitis, and lupus erythematosus. In this hemodialysis center, patients aged 51–60 and 61–70 years were majorly enrolled and had the highest levels of anxiety and depression.

In this study, the prevalence of anxiety and depression among hemodialysis patients was relatively high, 85.98, and 84.11%, respectively. This prevalence was higher than in other study that also used Beck Inventory to assess the depression and anxiety levels, where the prevalence rates of symptoms of depression and anxiety were 31.2 and 27.9%, respectively (de Brito et al., 2019). In another study that used Hospital Anxiety and Depression Scale (HADS), anxiety was recognized in 49.6% of the patients, while depression was identified in 55% of the cohort (Nagy et al., 2023). A similarity in the prevalence of anxiety and depression was apparent in two studies conducted in Saudi Arabia (83%) using the Zung self-rating depression scale, and in other parts of India using BDI (83.5%) (AlDukhayel, 2015; Nelson et al., 2015).

In our study, men had higher levels of anxiety and depression than women, but there are no appreciable disparities between the genders. These findings are in line with earlier research that revealed no significant differences in the rate of mental disorders between men and women (Yoong et al., 2017; Kao et al., 2020; Kamel et al., 2022), but are inconsistent with other recent studies that indicated substantial differences (Gerogianni et al., 2018; Delgado-Domínguez et al., 2021; Nagy et al., 2023). We found an association between anxiety and patient age, more specifically in the 51–60 and 61–70 age groups; however, no association between age and depression was discovered. Furthermore, a correlation was discovered between anxiety disorders and nonsmokers, diabetes, arrhythmias, and arterial hypertension. Living with family, having an overweight BMI, and not smoking were found to be associated with depression. An association between anxiety and depression and time in dialysis of patients aged 6–10 years and >11 years was observed, as well as a correlation between anxiety and depression, and diabetes, arrhythmias, arterial hypertension or cardiovascular diseases, and in patients with a duration of diabetes for more than 5 years. These sociodemographic and clinical factors had a high impact on psychological disorders, potentially contributing to the high frequency of depression and anxiety among hemodialysis patients in our study.

As reported previously, in Albania 3.8, and 4.8% of the population suffer from anxiety disorders, and depression, respectively, (World Health Organization, 2017), however no data are available on the depression and anxiety levels in Albanian HD patients. Data have reported a prevalence of 348 cases per million of renal replacement therapy in Albania (Spasovski et al., 2019). Despite the main limitation of the current study, the small sample size that is not representative of the whole population, considering the very high levels of depression and anxiety reported in our study we hypothesize that the levels of depression and anxiety are significantly higher in this group of patients in confront to the general population. Some of the patients probably having anxiety and depression did not agree to be part of the study, hence the selection bias is another limitation of our study. Since no other similar study using BAI and BDI was ever performed in Albania, and the cut-off values for BAI and BDI were established based on different literature studies, the validity of BAI and BDI is a limitation of the study. The well established diagnosis is a strength of our study. In addition, other strength of the study is that in order to avoid any potential reporting errors patients were provided with continuous and repeated information on the respective questionnaires.

## 5 Conclusion

According to our findings, the prevalence of mental disorders (such as depression and anxiety) is significant among patients with renal failure on hemodialysis. Despite increased interest in the psychiatric diagnostic as a reliable indicator of psychopathology in ESRD populations, there is still more work to be done, particularly in our country. We recommend hemodialysis patients to undergo routine screening and to refer to psychological health specialists to examine mental health disorders with the primary goal of enhancing their quality of life. As a result, we propose that future research should incorporate other factors that evaluate hemodialysis effectiveness and assess the link between these factors and mental health conditions. The proper management of some of these factors may influence patient outcomes in terms of a lower score of depression and anxiety, and would significantly improve the lives of patients.

## Data availability statement

The raw data supporting the conclusions of this article will be made available by the authors, without undue reservation.

## Ethics statement

Ethical approval was obtained by the University of Elbasan “Aleksander Xhuvani” as part of the research on “Quality of life in hemodialysis patients. Written informed consent to participate in this study was not required from the participants or the participants’ legal guardians/next of kin in accordance with the national legislation and the institutional requirements”.

## Author contributions

BE: Conceptualization, Data curation, Formal analysis, Investigation, Methodology, Project administration, Resources, Supervision, Validation, Visualization, Writing – original draft, Writing – review & editing. EA: Data curation, Formal analysis, Software, Writing – review & editing. BZ: Data curation, Funding acquisition, Visualization, Writing – review & editing. MH: Data curation, Resources, Supervision, Validation, Visualization, Writing – original draft, Writing – review & editing.
